# Cost and operational context for national human papillomavirus (HPV) vaccine delivery in six low- and middle-income countries

**DOI:** 10.1016/j.vaccine.2023.11.008

**Published:** 2023-11-30

**Authors:** Mercy Mvundura, Rose Slavkovsky, Frédéric Debellut, Teddy Naddumba, Amare Bayeh, Cathy Ndiaye, Jacqueline Anena, Elisabeth Vodicka, Abdou Diop, Deepa Gamage, Clarisse Musanabaganwa, Ganesh Tatkan, Alfred Driwale, Meseret Zelalem, Ousseynou Badiane, Samitha Ginige, Ertenisa Hamilton, Hassan Sibomana, Yohannes Lakew, Francois Uwinkindi, Adugna Dhufera, Immaculate Ampeire, Sandeep Kumar, D. Scott Lamontagne

**Affiliations:** a PATH, Seattle, WA, USA; b PATH, Geneva, Switzerland; c PATH, Kampala, Uganda; d PATH, Addis Ababa, Ethiopia; e PATH, Dakar, Senegal; f Ministry of Health, Colombo, Sri Lanka; g Rwanda Biomedical Center, Kigali, Rwanda; h Ministry of Health, Primary Health Care, Georgetown, Guyana; i Uganda National Expanded Programme on Immunization, Kampala, Uganda; j Federal Ministry of Health, Addis Ababa, Ethiopia; k Ministry of Health, Dakar, Senegal; l Ethiopia Public Health Institute, Addis Ababa, Ethiopia; m PATH, New Delhi, India; n JSI Research & Training Institute, Inc., Arlington, TX, USA^1^

**Keywords:** Human papillomavirus vaccine, Delivery costing, Microcosting, Vaccine economics, Immunization costing, Operational research, Low- and middle-income countries

## Abstract

**Introduction:**

There are concerns from immunization program planners about high delivery costs for human papillomavirus (HPV) vaccine. Most prior research evaluated costs of HPV vaccine delivery during demonstration projects or at introduction, showing relatively high costs, which may not reflect the costs beyond the pilot or introduction years. This study sought to understand the operational context and estimate delivery costs for HPV vaccine in six national programs, beyond their introduction years.

**Methods:**

Operational research and microcosting methods were used to retrospectively collect primary data on HPV vaccination program activities in Ethiopia, Guyana, Rwanda, Senegal, Sri Lanka, and Uganda. Data were collected from the national level and a sample of subnational administrative offices and health facilities. Operational data collected were tabulated as percentages and frequencies. Financial costs (monetary outlays) and economic costs (financial plus opportunity costs) were estimated, as was the cost per HPV vaccine dose delivered. Costing was done from the health system perspective and reported in 2019 United States dollars (US$).

**Results:**

Across the study countries, between 53 % and 99 % of HPV vaccination sessions were conducted in schools. Differences were observed in intensity and frequency with which program activities were conducted and resources used. Mean annual economic costs at health facilities in each country ranged from $1,207 to $3,190, while at the national level these ranged from $7,657 to $304,278. Mean annual HPV vaccine doses delivered per health facility in each country ranged from 162 to 761. Mean financial costs per dose per study country ranged from $0.27 to $3.32, while the economic cost per dose ranged from $3.09 to $17.20.

**Conclusion:**

HPV vaccine delivery costs were lower than at introduction in some study countries. There were differences in the activities carried out for HPV vaccine delivery and the number of doses delivered, impacting the cost estimates.

## 1 Introduction

Cervical cancer is the fourth most common cancer in women globally with an estimated 342,000 deaths and 604,000 new cases in 2020 [1]. More than 85 % of the cervical cancer burden is in low- and middle-income countries (LMICs) [1]. Almost all cases of cervical cancer can be attributed to human papillomavirus (HPV). Vaccines that protect against HPV have been available for almost two decades, and the World Health Organization (WHO) recommends the introduction of HPV vaccine in all national immunization programs [2]. Gavi, the Vaccine Alliance has set the goal of immunizing 86 million girls by 2025 to avert an estimated 1.4 million deaths [3]. As of July 2023, approximately 55 % of LMICs had introduced HPV vaccines in their national immunization programs [4].

Concerns about costs, including for vaccine procurement and programmatic costs for vaccine delivery, are one of the reasons that more LMICs have not yet introduced HPV vaccination. Delivery costs still remain a concern for program planners and stakeholders in countries that have introduced as they consider long term program sustainability [5]. Existing research provides evidence that the cost to deliver HPV vaccines to adolescents is generally higher than for vaccines targeting infants [6]. This is partly due to lack of scale for HPV vaccines, as in most programs, only HPV vaccine is administered to the target cohort of adolescents, in contrast to infant routine immunization where multiple vaccines are administered to the cohort. Another reason for the relatively higher costs is because most HPV vaccination programs have leveraged school-based settings to administer the vaccine, which is a more expensive delivery strategy for routine vaccination, in contrast to infant vaccinations that are given mainly in facility-based settings at a lower operational cost [7].

Reported costs of HPV vaccine delivery are wide ranging, depending on costing methods, components included, country characteristics, delivery strategy, and other factors. Most prior research evaluated costs of HPV vaccine delivery during demonstration projects or the initial years of vaccine introduction [6], [8], [9], [10], [11], [12]. An evaluation of operational costs of HPV vaccine delivery during demonstration projects in 12 countries eligible for support from Gavi estimated average financial costs of $8.30 per dose and average economic costs of $13.28 (2014 US$) per dose when excluding the cost of vaccines and supplies [6].

Costing studies conducted for demonstration projects or during the first year of vaccine introduction may not reflect the costs beyond the pilot or introduction years. National HPV vaccination programs are classified as having a facility-based, school-based, or mixed strategy, however there is limited evidence comparing the national strategy with how HPV vaccines are delivered by the implementing health facilities. There is a dearth of evidence on how the operational context for HPV vaccine delivery differ across countries. Given this, our study aimed to address the limitations of the current evidence-base. Our objectives were to understand the contextual and operational factors of HPV vaccine delivery in six national immunization programs that are past the introduction years and to estimate their ongoing delivery costs, from the perspective of the health system.

## 2 Materials and methods

### 2.1 Study countries

To ensure a diverse representation of country experiences in HPV vaccine delivery, we used multiple criteria in selecting the study countries, as shown in the country characteristics in Table 1 [13], [14]. All countries included in this study received support from Gavi for national introduction of HPV vaccines.

**Table 1:** Study countries, characteristics and sample sizes.

	Ethiopia	Guyana	Rwanda	Senegal	Sri Lanka	Uganda
**Country characteristics**						
Month and year of nationwide HPV vaccine introduction	December 2018	January 2017	April 2011	October 2018	September 2017	October 2015
WHO region	AFRO	PAHO	AFRO	AFRO	SEARO	AFRO
HPV vaccine delivery strategy based on WHO classification [13]	School-based	School-based	School-based	Facility-based	Mixed	School-based
Gender and target age group for HPV vaccination (2019)	14-year-old girls	10-year-old girls and boys	12-year-old girls (grade 6)	9-year-old girls	10-year-old girls (grade 6)	10-year-old girls
Number of doses in the HPV vaccine schedule (2019)	2 doses	2 doses	2 doses	2 doses	2 doses	2 doses
HPV vaccine interval between doses (2019)	6 months	6 months	6 months	6 months	6 months	6 months
Reported national target population size for HPV vaccination (2019)	1,284,036	15,000	149,111	204,235	173,130	681,758
National coverage for HPV vaccine last dose, official coverage (2019) [14]	94 %	55 % females; 62 % males	97 %	27 %	99 %	65 %
**Sample sizes**						
Health facilities	60	43	42	56	30	66
District administrative offices	17	n/a	11	14	10	21
Zones or sub-cities administrative offices	9	n/a	n/a	n/a	n/a	n/a
Regional administrative offices	3	5^†^	n/a	7	n/a	n/a
National administrative office	1	1	1	1	1	1

Abbreviations: AFRO: Africa region of the World Health Organization; HPV: human papillomavirus; PAHO: Pan American Health Organization; Americas region of the World Health Organization; SEARO: South-East Asia region of the World Health Organization; WHO: World Health Organization.

Note: Fields indicated not applicable (n/a) when the administrative level is not included in the country or does not have a role in immunization program activities. ^†^In Guyana, two administrative offices were interviewed in one region, resulting in five observations at the subnational level in four regions.

### 2.2 Study design

This was a cross-sectional, retrospective, mixed-methods study utilizing implementation science methods and microcosting approaches, aligned with the methodological guidelines for operations research of HPV vaccine program context [15] and costing of immunization programs [16]. The operations research component aimed to describe program context and implementation factors for the HPV vaccination program activities. It evaluated what program activities were conducted, how each activity was done and how often, and who was involved. The study evaluated 11 HPV vaccine program activities: vaccine procurement; estimating demand; program planning and management; social mobilization and information, education, and communication (IEC); training; vaccine collection or distribution and storage; service delivery; supervision; record keeping; waste management; and crisis management. The costing research component identified and quantified the relevant resources used for each of these HPV vaccination program activities in 2019. Costs were evaluated from the health system perspective with no tracking of the payor.

### 2.3 Study sample sizes

The study sample was selected through a two-step process, with selection based on geography or socioeconomic characteristics to determine regions/provinces in each country. This was followed by stratified random sampling that identified health facilities and other subnational administrative levels. The sample selection for health facilities and subnational administrative levels used the EPIC Sample Design Optimizer tool [17], informed by secondary data obtained from national immunization program administrative databases. Health facilities were weighted proportional to target population size, with the number of eligible children for HPV vaccination in the catchment area used as a proxy for size. Table 1 shows the final sample sizes for each country.

### 2.4 Data collection

Primary data collection was conducted during a maximum of a four-week period in each country, between April 2021 and June 2022. Four weeks was adequate given the sample size and the size of the data collection team. Ad hoc, follow-up data collection activities in each country were conducted as data analysis identified remaining gaps.

At health facilities, a structured questionnaire was used to interview staff working on HPV vaccination program activities. Table A1 in the appendix shows the key data points collected for the operations research and costing. Extraction of data from health facility records such as tally sheets and vaccination session reports was done to capture information on each HPV vaccination session, including location (e.g., health facility, school, or other), session date, and number of vaccine doses delivered. At the administrative levels, immunization program managers were interviewed using a similar questionnaire adapted for the level of the health system. Data collection was done electronically on tablets using Open Data Kit software [18].

Secondary data on unit prices such as salary scales for staff working in the health and education sectors, replacement prices for equipment and vehicles, etc., were obtained from government documents (see Table A2 in the appendix). In addition, secondary data on quantities of HPV vaccine doses and infant vaccines used at administrative levels during the reference period were obtained from the immunization program databases.

### 2.5 Operations research data analysis

The operational data were analyzed using software including Excel (Microsoft Corporation, Redmond, Washington, USA) and SPSS (IBM Corporation, Armonk, New York, USA) and tabulated to provide information on the context for HPV vaccine delivery at each facility or administrative-level office. Counts and means of the continuous variables were computed. For categorial variables, frequencies were tabulated. The extracted data on HPV vaccination sessions and doses used at health facilities were analyzed using SAS Studio (SAS Institute Inc., Cary, North Carolina, USA) to provide information on number of sessions by delivery location and doses delivered during the reference period.

### 2.6 Costing data analysis

This study defines ongoing delivery costs as the annual HPV vaccination program costs after the introduction years. Both the financial and economic costs were included: Financial costs are concerned with accounting transactions (monetary outlays or expenditures). Financial costs include per diems; costs for hosting meetings including venue rentals, food, etc.; vehicle rental costs and costs for riding public transport; fuel for vehicles and equipment; costs for developing or disseminating social mobilization contents such as radio messages and printed materials; shipping, handling, and customs clearance costs for vaccines and supplies; and other expenditures such as copying and printing record keeping materials. Economic costs combine financial costs with opportunity costs, which represent the value of using existing resources when a direct financial outlay is not incurred by the HPV vaccination program.^17^ Opportunity costs include costs for health worker and non-health worker time (ministry of education staff, community stakeholders, and volunteers) and annualized costs of using existing vehicles and equipment (cold chain, incinerators, etc.).

Cost data were collected in local currency and converted to 2019 United States dollars (US$) using the average World Bank exchange rate for 2019. Costing data analysis was done using Stata (StataCorp LLC, College Station, TX, USA) following costing methodological guidelines [15]. Data were analyzed disaggregated by the level of the health system, with the health facility or administrative-level office as the unit of analysis. To obtain cost estimates for HPV vaccination program activities, the quantities of each resource used were multiplied by their unit price or opportunity cost. For capital items (e.g., vehicles, equipment), we annualized the replacement price over their assumed useful life-year using a 3 % discount rate. Where values were reported as unknown by a facility in the sample, missing data were imputed using the median value of responses given by other facilities in the sample. Shared costs were allocated to the HPV vaccination program based on the reported proportions in the questionnaire or the quantity and volume-based proportions calculated using the number of HPV vaccine doses delivered among the total doses delivered for infant vaccines.

All cost estimates are reported excluding the value of vaccines and supplies. Weighted-mean costs per health facility or administrative office are reported, as well as the 95 % confidence intervals. A volume-weighted mean cost per HPV vaccine dose was calculated using the following formula:

$$\mathrm{Volume}\;\mathrm{weighted}\;\mathrm{mean}\;\mathrm{cost}\;\mathrm{per}\;\mathrm{dose}=\frac{\sum_{i=1}^{n}{\mathrm{Cost}}_{i}}{\sum_{i=1}^{n}{\mathrm{Doses}}_{i}}$$

where *i* is each site in the study sample at that level of the health system and *n* is the sample size for that level of the health system. At the health facility level, cost per dose was calculated using the extracted HPV vaccine dose data as the denominator, except in Uganda where these data are from the health information system due to incomplete records at study facilities. Cost per dose is calculated for administrative levels using data on doses delivered within its catchment area based on administrative data. Finally, the total mean cost per dose was computed, aggregated across all levels of the health system.

### 2.7 Ethics reviews

The study was determined to be exempt from US-based institutional review board (IRB) oversight. In Senegal, the study was considered program evaluation by the Ministry of Health (MOH). The Guyana MOH IRB waived the protocol from review. The study was approved by the Ethiopian Public Health IRB, Rwanda National Ethics Committee, National Hospital of Sri Lanka Ethics Review Committee, and Makerere University School of Public Health Research Ethics Committee (Uganda) and Uganda National Council for Science and Technology.

## 3 Results

### 3.1 HPV vaccine delivery program context

In 2019, HPV vaccines were primarily (>70 % of sessions) delivered at school-based vaccination sessions in five of the six study sites except in Senegal, where just over half of HPV vaccinations sessions were conducted in schools (Table 2). Some countries also provided HPV vaccine at health facilities and/or through non-school-based outreach sessions in the community. The average number of doses delivered in HPV vaccination sessions varied widely across the six countries. The average number of doses delivered per health facility was larger in Sri Lanka and Rwanda, reflecting their near-exclusive use of school locations where large numbers of girls would be present. Ethiopia, Rwanda, and Uganda delivered HPV vaccines primarily during two fixed points in time over the year. In the other three countries, vaccination sessions were conducted during all months of the year.

**Table 2:** HPV vaccine program operational context at health facilities in the study sample in 2019.

Activity	Variable	Ethiopia (n = 60)	Guyana (n = 43)	Rwanda (n = 42)	Senegal (n = 56)	Sri Lanka (n = 30)	Uganda (n = 66)
Service delivery	Number (%) of health facilities in the study sample providing HPV vaccination services in reference year	51 (85 %)†	40 (93 %)	41 (98 %)	55 (98 %)	30 (100 %)	52 (79 %)
	Number (%) of HPV vaccination sessions by location:						
	Schools	191 (89 %)	153 (71 %)	386 (99 %)	319 (53 %)	733 (95 %)	258 (78 %)
	Health facilities (on health facility site)	7 (3 %)	54 (25 %)	1 (<1 %)	194 (32 %)	36 (5 %)	15 (5 %)
	Outreach (non-school based)	16 (7 %)	9 (4 %)	1 (<1 %)	91 (15 %)	N/A	58 (18 %)
	Average number of HPV vaccine doses delivered per vaccinating health facility	411	170	613	212	761	162
	Average number of HPV vaccination sessions held per vaccinating health facility in 2019	4.0	5.4	9.5	11.0	25.6	6.4
	Timing of HPV vaccination sessions in the study sample	Twice per year, fixed months	Continuous throughout the year (1 peak)	Twice per year, fixed months	Continuous throughout the year	Continuous throughout the year (2 peaks)	Continuous throughout the year (2 peaks)
Program planning and management	Number (%) of HF reporting conducting the activity	47 (78 %)	27 (63 %)	34 (81 %)	50 (89 %)	30 (100 %)	42 (64 %)
	Average number of activities conducted per health facility when activity was done	3.2	6.7	2.7	6.1	13.1	3.6
Social mobilization and IEC	Number (%) of HF reporting conducting the activity	45 (75 %)	36 (84 %)	25 (60 %)	56 (100 %)	23 (77 %)	49 (74 %)
	Average number of activities conducted per health facility when activity was done	5.4	4.8	2.6	5.6	15.6	6.7
Training	Number (%) of HF reporting conducting the activity	35 (58 %)	9 (21 %)	4 (10 %)	36 (64 %)	11 (37 %)	20 (30 %)
	Average number of activities conducted per health facility when activity was done	3.4	1.6	1.5	1.5	2.0	3.1
Crisis management	Number (%) of HF reporting conducting the activity	19 (32 %)	13 (30 %)	2 (5 %)	34 (61 %)	5 (17 %)	10 (15 %)
	Average number of activities conducted per health facility when activity was done	1.3	1.6	1.0	2.4	1.4	1.3
Vaccine collection or distribution and storage	Number (%) collecting vaccines from higher-level facilities	17 (28 %)	23 (53 %)	42 (100 %)	44 (79 %)	2 (7 %)	41 (62 %)
	Average number of trips made to collect HPV vaccines (and other vaccines if combined trips) per health facility when activity was done	2.3	5.9	2.5	7.4	2.0	10.0
	Number (%) of HF with refrigerators for storing HPV vaccines (and other vaccines)	43 (72 %)	31 (72 %)	42 (100 %)	53 (95 %)	30 (100 %)	60 (91 %)
Waste management	Number (%) of health facilities conducting waste management activities on site	49 (82 %)	11 (26 %)	23 (55 %)	3 (5 %)	0 (0 %)	66 (100 %)
Supervision	Number (%) of HF receiving at least one supervision visit	53 (88 %)	30 (70 %)	38 (90 %)	53 (95 %)	27 (90 %)	57 (86 %)
Estimating demand	Number (%) of HF reporting knowing the total eligible population for HPV vaccination	58 (97 %)	32 (74 %)	42 (100 %)	56 (100 %)	30 (100 %)	56 (85 %)
Vaccine procurement	Number (%) of HF reporting they requested vaccines just before dose administration (otherwise had existing stock)	51 (100 %)†	11 (26 %)	42 (100 %)	5 (9 %)	2 (7 %)	5 (8 %)
Record keeping	Number (%) of HF reporting that they collect and maintain HPV vaccine session data	37 (62 %)	36 (84 %)	42 (100 %)	56 (100 %)	30 (100 %)	62 (94 %)

Abbreviation: HF: health facilities; HPV: human papillomavirus; IEC: information, education, and communication materials.

† Some health facilities in Ethiopia reported that they provided a supporting role to health posts and did not conduct HPV vaccination directly; 51 health facilities conducted HPV vaccination directly.

Table 2 shows the frequency with which other HPV vaccination program activities were conducted and the average number of times they were done by facilities engaging in these activities. Across all countries, between 63 % (Guyana) and 100 % (Sri Lanka) of health facilities in the study sample held program planning meetings and between 60 % (Rwanda) and 100 % (Senegal) conducted social mobilization activities. In Ethiopia and Senegal, at least 58 % of the health facilities sampled reported that staff attended HPV vaccine-related training activities, but in the other study countries, less than 40 % of the health facilities reported participation in training. In Senegal, 61 % of the health facilities reported conducting crisis management response activities, which is higher than in the other five study countries, where crisis management activities were conducted by up to a third of the health facilities. In all six countries, there were no suspected serious adverse events following immunization (AEFI) with HPV vaccines, as reported in the interviews. Crisis responses regarding HPV vaccine were mostly to address rumors around fertility and safety.

At least 73 % of the subnational administrative offices in the sample reported conducting program planning activities (Table 3). Social mobilization was done by a high proportion (≥86 %) of the subnational offices in four of the study countries, but in Rwanda and Sri Lanka, only 18 % and 10 % of offices, respectively, did social mobilization. No training activities were conducted at the subnational administrative level in Rwanda, but training was done by 71 % and 62 %, of the subnational offices in Ethiopia and Senegal, respectively. No crisis management activities were conducted in two of the study countries (Rwanda and Sri Lanka) at the subnational administrative level. At least 60 % of the subnational administrative levels reported conducting supervision visits to health facilities.

**Table 3:** HPV vaccine delivery operational context at subnational administrative level offices in the study sample in 2019.

Activity	Variable	Ethiopia (n = 29)	Guyana (n = 5)	Rwanda (n = 11)	Senegal (n = 21)	Sri Lanka (n = 10)	Uganda (n = 21)
Program planning and management	Number (%) of administrative offices reporting conducting the activity	27 (93 %)	5 (100 %)	8 (73 %)	18 (86 %)	9 (90 %)	20 (95 %)
	Average number of activities conducted per administrative office when activity was done	3.7	9.8	3.6	6.4	10.4	4.2
Social mobilization and IEC	Number (%) of administrative offices reporting conducting the activity	28 (97 %)	5 (100 %)	2 (18 %)	19 (90 %)	1 (10 %)	18 (86 %)
	Average number of activities conducted per administrative office when activity was done	2.0	4.6	2.5	2.5	1.0	7
Training	Number (%) of administrative offices reporting conducting the activity	21 (72 %)	3 (60 %)	0 (0 %)	13 (62 %)	4 (40 %)	8 (38 %)
	Average number of activities conducted per administrative office when activity was done	4.9	14.8	n/a	1.9	7.0	2.1
Crisis management	Number (%) of administrative offices reporting conducting the activity	10 (34 %)	3 (60 %)	0	14 (67 %)	0	6 (29 %)
	Average number of activities conducted per administrative office when activity was done	1.6	1.4	n/a	1.5	n/a	1.2
Vaccine collection or distribution and storage	Number (%) of administrative offices collecting vaccines from higher-level facilities	9 (31 %)	2 (40 %)	8 (73 %)	12 (57 %)	4 (40 %)	3 (14 %)
	Number (%) of administrative offices delivering vaccines to lower-level facilities	12 (41 %)	3 (60 %)	0 (0 %)	10 (48 %)	10 (100 %)	14 (67 %)
Waste management	Number (%) of administrative offices conducting waste management activities	10 (34 %)	3 (60 %)	8 (73 %)	15 (71 %)	0 (0 %)	4 (19 %)
Supervision	Number (%) of administrative offices conducting supervision visits	28 (97 %)	5 (100 %)	11 (100 %)	21 (100 %)	6 (60 %)	18 (86 %)
Estimating demand	Number (%) of administrative offices reporting knowing the total eligible population for HPV vaccination	29 (100 %)	4 (80 %)	11 (100 %)	21 (100 %)	10 (100 %)	20 (91 %)
Vaccine procurement	Mean number of HPV vaccine doses delivered during the reference period (2019 vaccination cohort)	District: 2,132					
		Zone or sub-city: 26,356	Region: 5,052 (mean per region in sample: 4,450)	District: 7,446	District: 5,414	District: 11,354	District: 11,154
		Region: 197,174	National: 29,493	National: 292,892	Region: 28,170	National: 314,815	National: 1,035,269
		National: 2,277,484			National: 291,454		
Record keeping	Number (%) of subnational program offices that conducted record keeping activities	26 (90 %)	5 (100 %)	9 (82 %)	15 (71 %)	10 (100 %)	15 (71 %)

Abbreviations: HPV; human papillomavirus; IEC: information, education, and communication materials.

National administrative level program activity results are not shown since N = 1. The national program office in Rwanda reported conducting social mobilization, but in Senegal and Sri Lanka, the national offices did not conduct this activity. No training activities were conducted at the national level in Rwanda, Sri Lanka, and Senegal. Only Ethiopia reported conducting crisis management activities at the national level, in response to suspected serious AEFIs. All national level offices except in Senegal conducted supervision visits to lower administrative levels including health facilities.

### 3.2 Program costs for HPV vaccine delivery

The weighted-mean annual financial costs for HPV vaccine delivery per health facility were estimated to range from $164 (Guyana) to $497 (Senegal) and economic costs ranged from $1,212 (Uganda) to $3,190 (Sri Lanka) (Table 4). Across the study countries, financial costs were a smaller share of the economic costs at health facility level (ranging from 6 % to 35 %). At the administrative levels, there was a wide range in the mean financial cost estimated across the study countries, with the largest financial cost at the national level estimated for Uganda ($297,483), where activities were done as part of an HPV vaccine coverage improvement strategy. Some countries, such as Senegal and Sri Lanka, had very low financial and opportunity costs at the national level, with financial costs below $2,000 and economic costs below $12,000 in these two countries. In some of the study countries, financial costs were a large share of the economic costs at the administrative levels, such as in Ethiopia, where financial costs accounted for at least 76 % of the mean economic costs at the zone, region, and national levels. However, in other study countries, such as in Sri Lanka, opportunity costs were the larger share of costs even at the administrative levels, accounting for at least 75 % of the mean economic costs.

**Table 4:** Weighted mean and 95 % confidence intervals for financial and economic costs for HPV vaccine delivery at each level of the health system (in 2019 US$).

	Financial costs						Economic costs					
	Ethiopia	Guyana	Rwanda	Senegal	Sri Lanka	Uganda	Ethiopia	Guyana	Rwanda	Senegal	Sri Lanka	Uganda
**Annual costs**												
Health facility	$421 [$0–$975]	$164 [$89–$239]	$218 [$142–$295]	$497 [$231–$762]	$189 [$124–$255]	$423 [$149–$697]	$1,550 [$722–$2,375]	$1,979 [$878–$3,080]	$1,082 [$698–$1,466]	$2,169 [$1,372–$2,966]	$3,190 [$2,019–$4,360]	$1,212 [$653–$1,771]
District	$678 [$267–$1,088]	n/a	$304 [$137–$472]	$4,380 [$2,278–$6,482]	$459 [$190–$729]	$9,296 [$3,809–$14,783]	$4,833 [$273–$9,441]	n/a	$1,277 [$889–$1,665]	$7,313 [$3,700- $10,926]	$1,807 [$843–$2,770]	$11,007 [$4,457–$17,557]
Zone or sub-cities	$15,503 [$6,270–$37,277]	n/a	n/a	n/a	n/a	n/a	$18,485 [$3,427–$40,396]	n/a	n/a	n/a	n/a	n/a
Region	$44,592	$1,016	n/a	$2,950 [$754–$5,147]	n/a	n/a	$58,604	$12,570	n/a	$8,685 [$3,074– $14,296]	n/a	n/a
National office	$86,751	$25,012	$149,866	$1,930	$1,564	$297,483	$96,840	$60,944	$161,219	$11,568	$7,657	$304,278
**Cost per dose**												
Health facility	$1.05 [$0–$2.31]	$1.05 [$0–$2.29]	$0.48 [$0.29–$0.67]	$2.35 [$0.99–$3.70]	$0.22 [$0.13–$0.31]	$2.20 [$0.51–$3.89]	$3.88 [$2.19–$5.57]	$12.64 [$7.92–$17.37]	$2.37 [$1.68–$3.05]	$10.24 [$6.52–$13.96]	$3.70 [$2.56–$4.85]	$6.30 [$2.61–$9.99]
District	$0.32 [$0.07–$0.57]	n/a	$0.04 [$0.01–$0.07]	$0.81 [$0.32–$1.30]	$0.04 [$0.01–$0.07]	$0.83 [$0.24–$1.43]	$2.27 [$0.13–$4.66]	n/a	$0.17 [$0.09–$0.26]	$1.35 [$0.53–$2.17]	$0.16 [$0.09–$0.23]	$0.99 [$0.26–$1.71]
Zone or sub-cities	$0.59 [$0.08–$1.26]	n/a	n/a	n/a	n/a	n/a	$0.70 [$0.01–$1.42]	n/a	n/a	n/a	n/a	n/a
Region	$0.23	$0.20	n/a	$0.10 [$0–$0.21]	n/a	n/a	$0.30	$2.49	n/a	$0.31 [$0.03–$0.59]	n/a	n/a
National office	$0.04	$0.85	$0.51	$0.01	$0.01	$0.29	$0.04	$2.07	$0.55	$0.04	$0.02	$0.29
Total mean cost per HPV vaccine dose delivered aggregated across all levels of the health system	$2.23	$2.10	$1.03	$3.27	$0.27	$3.32	$7.19	$17.20	$3.09	$11.94	$3.88	$7.58

Abbreviations: HPV: human papillomavirus.

Note: Confidence intervals included only for health system levels where corresponding sample size was ≥7.

The mean financial cost per dose aggregated across all levels of the health system ranged from $0.27 (Sri Lanka) to $3.32 (Uganda), and the economic cost per dose ranged from $3.09 (Rwanda) to $17.20 (Guyana), as shown in Table 4. Across all study countries, health facilities contributed the largest share to the financial and economic cost per dose estimates, with between 47 % and 81 % of the aggregated financial cost per dose being borne at the health facility level. In addition, opportunity costs were the larger share of economic costs across all study countries.

There was no program activity that consistently accounted for the largest share of expenditures across all study countries; however, service delivery contributed to a large share of spending in all countries (see Fig. A1 in the appendix).

**Fig. 1:**
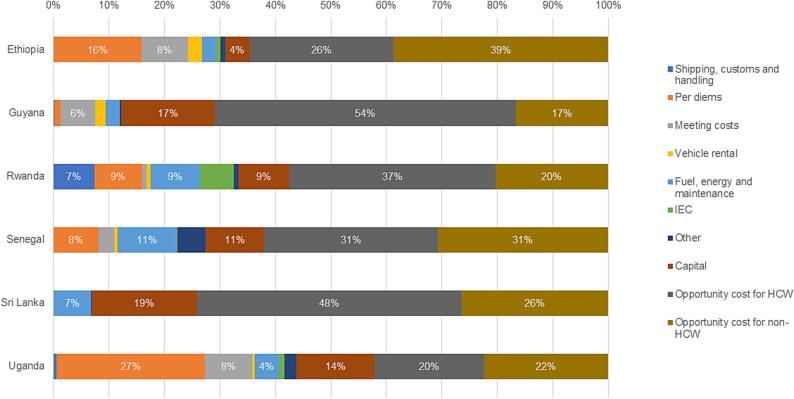
Proportion of the aggregated mean economic cost per dose by cost type.

Fig. 1 shows the proportion of cost types in the aggregated mean economic cost per dose. Per diems were not paid in Sri Lanka. In Guyana, per diems accounted for only 1 % of the aggregated economic cost per dose, while in other study countries they accounted for between 8 % (Senegal) and 27 % (Uganda) of the economic cost per dose. Across all six countries, financial costs accounted for at most 44 % of the economic costs. The largest share of economic costs for HPV vaccine delivery across all levels of the health system were opportunity costs, mainly time for health workers and non-health workers involved in HPV vaccine delivery. These accounted for between 42 % (Uganda) and 74 % (Sri Lanka) of the economic costs.

## 4 Discussion

Our study is the first to present the operational context and estimated HPV vaccine delivery costs in six countries implementing nationwide vaccination programs. Our study findings provide evidence from the period just prior to the COVID-19 pandemic when momentum was building to increase program coverage and extend the reach of this lifesaving vaccine to eligible target populations [19]. The evidence we provide in this study can be used to inform program planning and decision making as the programs work to rebuild post-pandemic.

Our study found that service delivery locations varied, but school-based delivery was the predominant strategy for HPV vaccines across all study countries, similar to previous findings [7], [20], [21]. In Senegal, the predominant delivery strategy leveraged was school-based, different from the characterization of the program as facility-based [13]. We found that health facilities do not offer HPV vaccinations with the same frequency as infant vaccines, which are offered daily, weekly, or monthly. Rather, most health facilities in the study countries conducted a few HPV vaccination sessions during the year, in hopes of having larger session sizes [7]. We also found differences in the number of months HPV vaccines were offered, with programs in Ethiopia and Rwanda conducting sessions during fixed time points of the year while other programs leveraged a year-round approach. Also, for several of the study countries, HPV vaccine is given in outreach and/or facility-based settings in addition to schools, providing multiple opportunities for adolescents to get vaccinated.

Our study also found that HPV vaccination programs are engaged in multiple activities that provide the supportive and administrative architecture to service delivery. The frequency and intensity of the activities differed across countries. In the study countries, the frequency of some program activities declined, depending on the years passed since HPV vaccine introduction, but some activities remained. Rwanda had the longest-running HPV vaccination program in our study sample (introduced in 2011), and we observed that training and crisis management activities were either not done or done by very few health facilities and administrative level offices. However, social mobilization was still conducted, mainly at the national level through a radio program, as new cohorts are targeted for vaccination each year.

In all study countries, we found that the financial cost per dose was lower than the opportunity cost per dose, indicating that existing resources are the largest resource for HPV vaccine delivery. Other studies have reported similar findings [6], [8], [11]. While all the countries included in our study received Gavi support for their HPV vaccine introduction, we note that Sri Lanka did not pay per diems for HPV vaccine program activities, even at introduction. This finding may imply that countries can tailor their expenditures to suit their context, even when supported by Gavi for introduction activities.

Our study also found that, when the cost per dose is aggregated across all levels of the health system, health facilities contributed the larger share of both financial and economic cost per dose compared with the administrative levels. The absolute costs per facility at the health facility level are lower than at administrative levels and so are the number of doses delivered, but the latter outweighs the former, thus increasing the cost per dose estimate at the health facility level.

Service volume, as measured by HPV vaccine doses delivered, is inversely related to cost per dose, and we observed a wide range in the mean number of HPV vaccine doses delivered per health facility in the study countries. Expanding service volume through larger session sizes, increased coverage, or strategies such as vaccinating multi-age cohorts could reduce a facility’s cost per dose.

Our study found that no single activity consistently contributed to the largest share of costs at subnational administrative levels. Although the same program activities may be done across countries, the frequency and intensity of the activities differed and, as a result, so did the relative spending on the activities. In comparing our study results with prior HPV vaccine costing studies in the same countries, cost per dose declined after the pilot or introduction period in Ethiopia and Rwanda. Estimates based on Ethiopia’s demonstration pilot in 2015/2016 found financial and economic cost per dose was $3.92 and $6.97 [22], respectively (equivalent to $4.19 and $7.46, respectively, in 2019 US$). Similarly, the financial and economic cost per dose estimates from a study during HPV vaccine introduction in Rwanda were $3.37 and $4.76 (in 2012 US$) [23], respectively (equivalent to $3.76 and $5.31, respectively, in 2019 US$).

Conversely, findings from a prior study in Senegal conducted at introduction estimated the financial and economic cost per dose at $3.07 and $7.56 (in 2020 US$) [24], respectively (equivalent to $2.97 and $7.32, respectively, in 2019 US$). Our cost estimates are higher, which could be partly due to fewer doses delivered in our study. Our findings are also higher than previous estimates from Uganda, which reported an average financial cost per dose of $2.10 and an economic cost per dose of $3.15 in 2009 US$ for school-based delivery [11] (equivalent to $2.49 and $3.74, respectively, in 2019 US$). This increase in cost may be due to the nationwide HPV vaccination coverage improvement campaign conducted during the reference period for our analysis.

Compared to findings from studies conducted at introduction in other LMICs, our financial cost estimates are lower than those reported in a study of 12 Gavi-eligible countries, which estimated average financial costs per dose of $8.30 (2014 US$) [6]. Similarly, our estimated financial costs are lower than estimates from Mozambique and Zambia [10], [25] but within the range of costs estimated in Tanzania [26]. Differences in costing methods (microcosting versus costing tools), activities costed, country context, number of doses delivered, and other factors may explain some of these differences.

Our study has several limitations. Due to the COVID-19 pandemic, data collection was done at least two to three years after the HPV vaccination activities were conducted, subjecting our findings to recall bias. Our sampling was done proportionate to the size of the target population and so this may have biased the sample towards larger facilities. There were challenges with the availability and/or completeness of health facility records, which may have resulted in an underestimation of doses delivered and an overestimation of the costs per dose. This challenge was especially noted in Uganda, where dose numbers extracted from health facility records were much lower than those reported in the health information management system. For Guyana, we used the same quantity- and volume-based proportions across all levels of the health system when allocating shared resources, as routine immunization vaccine stock data were not available to enable these calculations by sub-national level. Data on the number of adolescents vaccinated were incomplete in most countries and so we do not report the cost per child vaccinated or per fully vaccinated child. For Uganda, the study year was an atypical year when a coverage improvement campaign was conducted which entailed high cost and so may overestimate the ongoing costs for this program. All countries included in our study received support from Gavi for HPV vaccine introduction and so may not represent countries that introduce without Gavi support. Our analysis did not identify the determinants of cost differences across countries, but this can be explored in additional analyses where sample size allows. Lastly, our study is a cross-sectional study and cannot inform how program costs change over time or inform coverage improvement strategies.

## 5 Conclusions

Our study evaluated HPV vaccination program context and costs in six LMICs that have implemented nationwide routine HPV vaccine delivery. While other delivery strategies were also used, HPV vaccines were largely administered through school-based sessions in all six countries. There were differences in operational context across the study countries, with differences in the type and intensity of activities done. There is a wide range in the financial and economic cost per dose estimates across the six countries, with these delivery costs beyond introduction years lower than in the first year of introduction in some countries. Financial spending for HPV vaccine delivery was relatively low, and the larger share of resource use was opportunity costs of human resources time. The results of our study provide empirical evidence to local and international policymakers on the ongoing costs of HPV vaccine delivery programs and the activities contributing to these costs, and this information can be used to inform budgeting and planning for program sustainability.

## Declaration of competing interest

The authors declare that they have no known competing financial interests or personal relationships that could have appeared to influence the work reported in this paper.

## Data Availability

The raw data used for this analysis will be available on DavaVerse after the manuscript is accepted for publication.

## Appendix A Supplementary material

Supplementary data 1 Supplementary materials including additonal details on the costing and operational context evaluated, unit prices and additonal results.
