# Supplementary material for: Cost and operational context for national human papillomavirus (HPV) vaccine delivery in six low- and middle-income countries
Source: Vaccine. 2023 Nov 30;41(49):7435–43. doi: 10.1016/j.vaccine.2023.11.008 (PMC10697825; doi:10.1016/j.vaccine.2023.11.008)
Supplement: Supplementary data 1 — Supplementary materials including additonal details on the costing and operational context evaluated, unit prices and additonal results. [file mmc1.docx]

**Appendix**

Table A1. Summary of operational context components and economic costs included in the study, by HPV vaccine program activity.

| **Activity** | **Summary of operational context components used for the operations research analysis and costing** | **Economic costs included** | |
| --- | --- | --- | --- |
|  |  | **Financial costs** | **Opportunity costs** |
| Vaccine procurement | - Number of HPV vaccine shipments received and doses received from procurement agencies (N) - Amount paid for shipping, handling, and customs clearance for HPV vaccines and supplies (N) - Whether the facility or office procured additional immunization supplies for HPV vaccinations (H, S) | - Shipping, handling, and customs costs for HPV vaccines and supplies (N) - Amount spent on additional immunization supplies directly procured by the facility or administrative office (H, S) | - Health worker time spent on the activity (H, S, N)^†^ |
| Estimating demand | - Denominator source for eligible population for HPV vaccination (H, S, N) - Process for estimating HPV vaccine demand (H, S, N) - Relationship between area responsible for denominator estimation and that responsible for vaccine procurement (N) | - n/a | - Health worker time spent on the activity (H, S, N) |
| Program planning and management | - Whether staff participated in program planning and management activities (H, S, N) - Frequency and duration of microplanning and management activities, if done (H, S, N) - Annual microplanning process for HPV vaccine, as separate or combined with infant vaccines (H, S, N) - Whether MOE and school staff and other stakeholders were involved in program planning and management; if involved, the number of school staff and stakeholders involved and whether per diems were paid (H, S, N) | - Per diems and travel allowances paid (H, S, N) - Meeting costs (venue hire, catering, other costs) (H, S, N) | - Health worker time spent on the activity (H, S, N) - Non-health worker (i.e., ministry of education and school staff and other stakeholders) time spent on the activity (H, S, N)^‡^ |
| Social mobilization and IEC | - Whether staff participated in social mobilization activities (H, S, N) - Types of social mobilization activities conducted and for which HPV vaccine dose (H, S, N) - Frequency and intensity of social mobilization activities (H, S, N) - Whether MOE and school staff and other stakeholders were involved in social mobilization activities; if involved, the number of these staff and stakeholders involved and whether per diems were paid (H, S, N) - Annual HPV consent process: EPI and MOE staff involvement, time, frequency, and intensity (H) | - Per diems and travel allowances paid (H, S, N) - Meeting costs (venue hire, catering, other costs) (H, S, N) - Costs of producing and airing of TV and/or radio spots/other forms of media (H, S, N) - Printing of IEC materials (H, S, N) - Distribution costs of IEC materials (H, S, N) | - Health worker time spent on the activity (H, S, N) - Non-health worker time spent on the activity (H, S, N) |
| Training | - Whether staff participated in training activities (H, S, N) - Frequency and duration of training activities, if done (H, S, N) - Whether training was separate or combined with infant vaccines and proportion of time spent on HPV vaccines, if combined (H, S, N) - Whether MOE and school staff and other stakeholders were involved in training activities; if involved, the number of these staff and stakeholders involved and whether per diems were paid (H, S, N) | - Per diems and travel allowances paid (H, S, N) - Meeting costs (venue hire, catering, other costs) (H, S, N) - Production and printing of training materials (H, S, N) | - Health worker time spent on the activity (H, S, N) - Non-health worker time spent on the activity (H, S, N) |
| Vaccine collection or distribution and storage | - Whether HPV vaccine collection and distribution was separate or combined with that for infant vaccines (H, S, N) - Frequency of trips to collect or deliver HPV vaccines, staff involved, and whether per diems were paid (H, S, N) - Modes of transport used for trips to collect or deliver HPV vaccines (H, S, N) - Whether any MOH vehicles were stationed at the facility or office and the details on vehicle make and fuel type used (H, S, N) - Amount spent on fuel and maintenance of MOH vehicles for HPV vaccine program activities (H, S, N) - Availability of cold chain equipment used for storing HPV and infant vaccines, types and quantities of the equipment used, and energy sources (H, S, N) | - Per diems and travel allowances paid (H, S, N) - Cost of hired vehicles and public transport (H, S, N) - Fuel costs and vehicle maintenance (H, S, N) - Energy costs for cold storage (H, S, N) | - Health worker time spent on the activity (H, S, N) - Annualized cost for vehicles, refrigerators, and vaccine carriers allocated for HPV vaccines (H, S, N) |
| Service delivery | - Locations (facility, school, and community based) where HPV vaccines were administered (H) - HPV vaccine session details extracted from tally sheets and immunization program reports (H) - Detailed information for each location where HPV vaccination sessions were held including number of vaccinators, volunteers, and other human resources traveling to each site to administer vaccines, duration of each session, transport costs to travel to and from the vaccination locations, and whether per diems were paid (H) - Whether school staff were involved in HPV vaccination sessions at school, number of staff involved, and whether per diems were paid (H) - Changes in HPV vaccine delivery between the reference year and prior year, and reasons why (H) - Characterization of vaccinations at schools: special outreach (separate funds), routine outreach (no separate funds), or not considered outreach (H) | - Per diems and travel allowances paid (H) - Hired vehicles and public transport used to travel to vaccination locations (H) - Fuel costs and vehicle maintenance (H) | - Health worker time spent on the activity (H) - Non-health worker time spent on the activity (H) - Annualized cost for vehicles (H) |
| Supervision | - Whether supervision visits for HPV vaccine were received (H) - Frequency and intensity of supervision visits for HPV vaccinations (S, N) - Whether supervision visits were separate or combined with supervision for infant vaccines (S, N) - Type of staff conducting supervision visits, costs of transport for supervision visits, and whether per diems were paid (S, N) - Modes of transport used when traveling for supervision visits (S, N) - Whether MOE or other stakeholders were involved in supervision activities; if involved, number of these staff and stakeholders involved and whether per diems were paid (S, N) | - Per diems and travel allowances paid (S, N) - Hired vehicles and public transport used (S, N) | - Health worker time spent on the activity (H, S, N) - Non-health worker time spent on the activity (S, N) - Annualized cost for vehicles (S, N) |
| Record keeping | - Receipt (quantity and frequency) of data recording and reporting tools (e.g., tally sheets, session recording forms, monthly reporting forms, vaccination cards, vaccination registers) and availability (H, S) - Frequency and timing of use of data recording and reporting tools (H, S) - Reporting flow for vaccination sessions from session to facility to higher levels, and timeliness (H, S) | - Extra printing or copying costs for record keeping materials paid by the facility or office (H, S) | - Health worker time spent on the activity (H, S, N) |
| Waste management | - Methods for disposing of immunization program waste (H, S) - Frequency of waste management activities (H, S) - Whether there were any travel costs to collect waste from lower-level facilities and if done, the details of the trips including modes of transport used and frequency of these trips and whether per diems were paid (S) | - Per diems and travel allowances (S) - Fuel costs for incineration (H, S) - Hired vehicles and public transport (S) | - Health worker time spent on the activity (H, S) - Non-health worker time spent on the activity (S, N) - Annualized cost for incinerators (H, S) |
| Crisis management | - Annual crisis communication planning process: staff involved, time, intensity (H, S, N) - Whether there were any crises or rumors that necessitated a response activity; if activity was done, details on the staff involved, frequency of the activity, intensity, and whether per diems were paid (H, S, N) - Whether MOE and school staff and other stakeholders were involved in the crisis response activities; if involved, the number of staff and stakeholders involved and whether per diems were paid (H, S, N) - Whether there were any specific investigations of any suspected serious AEFI and, if done, the details on the staff involved, time spent, frequency, outcome, and post-investigation re-sensitization or communication activities done (H, S, N) - Current rumors and impact on HPV vaccine dose 1 or dose 2: uptake and coverage before and after (H, S, N) | - Per diems and travel allowances (H, S, N) - Other costs (e.g., meeting costs or additional printing of IEC materials) (H, S, N) | - Health worker time spent on the activity (H, S, N) - Non-health worker time spent on the activity (H, S, N) |

(H) = health facility level; (S) = subnational administrative level(s); (N) = national administrative level.

AEFI: adverse event following immunization; EPI: Expanded Programme on Immunization; HPV: human papillomavirus; IEC: information, education, and communication; MOE: Ministry of Education; MOH: Ministry of Health.

^†^At the health-facility level, the questionnaire disaggregated health worker time use by activity; however, at administrative levels (S, N), health worker time use was reported aggregated as a percentage of time spent for all HPV vaccination activities. Thus, health worker time costs are reported in aggregate at the administrative levels.

^‡^Non-health worker time included volunteers, Ministry of Education and school staff, and community stakeholders (e.g., community leaders, religious leaders, parents’ associations, etc.) as relevant to each activity.

Table A2. Unit prices in 2019 US$.

|  | **Ethiopia** | **Guyana** | **Rwanda** | **Senegal** | **Sri Lanka** | **Uganda** |
| --- | --- | --- | --- | --- | --- | --- |
| **Exchange rate (local currency unit per US$1)** | 32 | 208.5 | 899 | 586 | 179 | 3704.05 |
| **Energy prices** |  |  |  |  |  |  |
| Petrol price per liter | $0.72 | $1.03 | $1.17 | $1.12 | $0.79 | $1.12 |
| Diesel price per liter | $0.81 | n/a | $1.17 | $1.12 | $0.55 | $1.04 |
| Electricity price per kilowatt hour | $0.007 | $0.320 | $0.093 | $0.166 | $0.036 | $0.129 |
| **Equipment and vehicles** |  |  |  |  |  |  |
| Refrigerated truck | n/a | n/a | $96,143 | $198,929 | n/a | $52,287 |
| 4WD truck | $88,824 | $35,971 | n/a | $22,680 | $38,324 | $52,287 |
| Motorcycle | n/a | $3,597 | $3,424 | $5,062 | n/a | $4,050 |
| Three-wheeler | n/a | n/a | n/a | n/a | $4,168 | n/a |
| Cost of constructing a new incinerator | $8,234 | $20,000 (health facility level)  $40,000  (administrative levels) | $10,000 | $25,601 | n/a | $41,141 |
| **Allocation factors for HPV vaccine versus other vaccines** |  |  |  |  |  |  |
| Quantity-based proportion (mean and range)^†^ | 0.03  (0.01–0.08) | 0.21 | 0.05  (0.03–0.06) | 0.05  (0.00–0.27) | 0.06  (0.02–0.26) | 0.02  (0.01–0.03) |
| Volume-based proportion (mean and range) ^‡^ | 0.10  (0.03–0.23) | 0.48 | 0.08  (0.05–0.15) | 0.21  (0.01–0.69) | 0.25  (0.12–0.65) | 0.10  (0.04–0.14) |
| **Monthly salaries** |  |  |  |  |  |  |
| Health workers | $144–$385 | $322–$1,760 | $142–$993 | $101–$1,489 | $157–$811 | $61–$1,268 |
| School staff such as teachers and administrators | $78–$347 | $336–$1,109 | $255–$603 | $307–$488 | $261–$436 | $62–$222 |
| Other non-health worker salaries (valuation of time for volunteers) | $78 | $212–$336 | $84 | $101 | $70 | $1.35–$84 |

EPI: Expanded Programme on Immunization; HPV, human papillomavirus.

^†^Quantity-based proportions were used to allocate human resources time for vaccine collection and distribution activities. The proportions were calculated using the number of HPV vaccine doses delivered among the total doses delivered for routine EPI vaccines during the reference period.

^‡^Volume-based proportions were used to allocate the annualized capital and energy costs for equipment such as cold chain equipment and incinerators. The proportions were calculated using the volume in cubic centimeters (cm^3^) of HPV vaccines delivered among the volume of all routine EPI vaccines during the reference period.

Figure A1. Proportion of the aggregated mean financial costs per dose by HPV vaccination program activity.

Figure A1 shows how each of the HPV vaccination program activities contributed to mean financial costs per dose across all levels of the health system. There was no program activity that consistently accounted for the largest share of expenditures across all study countries. Service delivery was one of the top three activities constituting the largest share of spending, however, the proportion of financial costs per dose spent on service delivery varied by country, ranging from 10% (Guyana) to 80% (Sri Lanka). Excluding Sri Lanka where there was no financial spending for social mobilization, expenditures for social mobilization accounted for between 2% (Guyana) and 29% (Uganda) of the mean cost per dose in the other five countries. In Rwanda, Senegal, and Uganda, social mobilization was also one of the three activities with a larger share of spending (ranging from 19% to 29% in these three countries), while in Ethiopia, Guyana, and Uganda, training was an activity with a larger share of spending (ranging from 17% to 46% in these three countries).
